# Unveiling Enigma: Navigating the Diagnostic Labyrinth of an Atypical Medial Clavicular Enchondroma

**DOI:** 10.7759/cureus.55098

**Published:** 2024-02-27

**Authors:** Adarsh Jayasoorya, Sandeep Shrivastava, Ankit M Jaiswal, Hardik Patel, Rohan Chandanwale

**Affiliations:** 1 Department of Orthopaedics, Jawaharlal Nehru Medical College, Datta Meghe Institute of Higher Education and Research, Wardha, IND

**Keywords:** intraosseous ganglion cyst, cartilagenous tumor, paediatric age, benign tumors, enchondroma

## Abstract

Benign cartilaginous lesions called enchondromas usually appear in the long bones of the limbs. This case report, however, draws attention to an uncommon and unusual appearance of enchondroma near the medial end of clavicle. Because of the unusual location, the diagnostic process was very complex, which presented a challengefor the physicians. We provide the clinical, radiological and histological results that finally allowed for an accurate diagnosis. This example highlights the need of taking into account atypical location for benign lesions and highlights the necessity of a thorough diagnostic approach in unexpected clinical settings. Since the occurrence of clavicular enchondromas is a rare entity and can at times mislead the clinician, healthcare providers must be vigilant enough to guarantee a prompt and accurate diagnosis for timely intervention.

## Introduction

Amongst connective tissues, the bone is considered unique for its exceptional mineralisation mechanism. Calcium hydroxyapatite is the inorganic component of bone that renders its toughness and strength. In addition, this mineral stores 65% of the body's sodium and magnesium while acting as the main store for calcium (99%) and phosphate (85%) [1]. Bone tumours containing hyaline cartilage can develop on the surface of the bone or within the medullary cavity. Bones of endochondral origin can harbour enchondromas, benign tumours made of hyaline cartilage from the medullary cavity [2]. These growths are mostly single lesions in the centre of the metaphyseal tubular bone whereas juxtacortical or subperiosteal chondromas develop on the surface of the bone. Though they can also infrequently develop in the femur and humerus, enchondromas most frequently occur in the hand and foot bones [3,4].

Primitive phalangeal bone tumours are the most prevalent type of bone tumours in the hand. The distal phalanges, metacarpals, and middle phalanges are additional often-occurring locations. Enchondromatous tumours usually start and expand in childhood, originating from chondrocytes or cartilage of growth plate remnants that proliferate at first but then cease developing correctly, continuing into adulthood [5]. The little finger is the most usually affected digit, and these tumours are mostly observed in the third and fourth decades of life [6]. One distinctive feature of hand enchondromas is that, upon histological inspection, they may exhibit cellular atypia, bearing a resemblance to chondrosarcoma [7]. Chondrosarcoma can develop from enchondromas, which have the potential to be malignant [7]. On the other hand, enchondromas usually result in low-grade cancers that sporadically spread to other parts of the body. Diagnosing enchondromas that have abnormal traits and are located in unexpected places can be difficult. Enchondromas can mimic both benign and malignant diseases, especially chondrosarcoma, and are usually treated non-surgically.

## Case presentation

**Figure 1:**
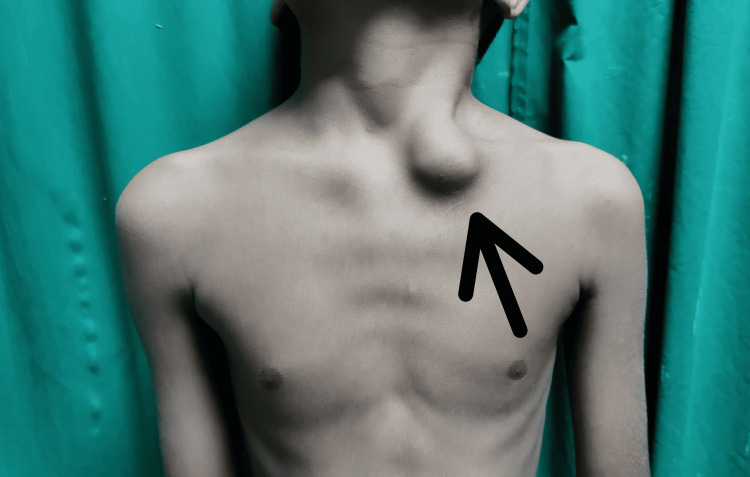
Clinical image showing a globular swelling over the medial end of clavicle

A 12-year-old boy presented to the orthopaedics outpatient clinic with a swelling on his upper left chest side that had persisted for one year at the time of presentation. The parents sought counsel after noticing a slow growth in the swelling over the past three months, which had grown from the size of a peanut to the size of a lemon. There was no prior history of fall or trauma, fever spikes, reduction in weight, or comparable swellings elsewhere in the body. Examining revealed normal-looking skin around the medial end of the clavicle (Figure 1), with no localised discomfort or temperature increase.

**Figure 2:**
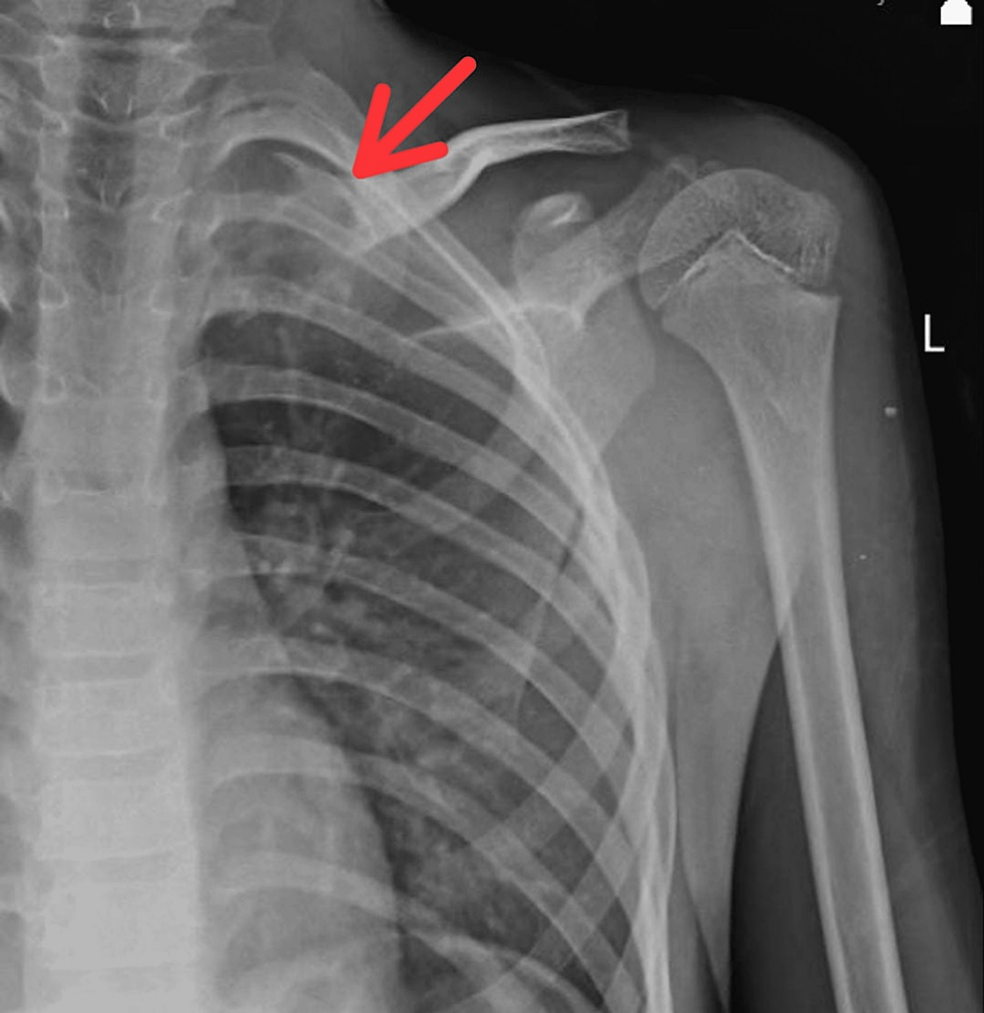
Pre-operative X-ray of the left shoulder with clavicle showing lesion over medial end of clavicle

A solitary, 3 × 3 cm globular enlargement was observed over the medial part of the clavicle. When the swelling was palpated, it felt hard and the distal neurovascular status was intact. The neck and shoulder movements were full and normal. Results from blood tests including complete blood count, erythrocyte sedimentation rate, C- reactive protein, and alkaline phosphates were normal. An approximately 3 x 3 cm lytic lesion with a sclerosed edge was visible over the medial end of the clavicle in the pre-operative X-ray (Figure 2).

**Figure 3:**
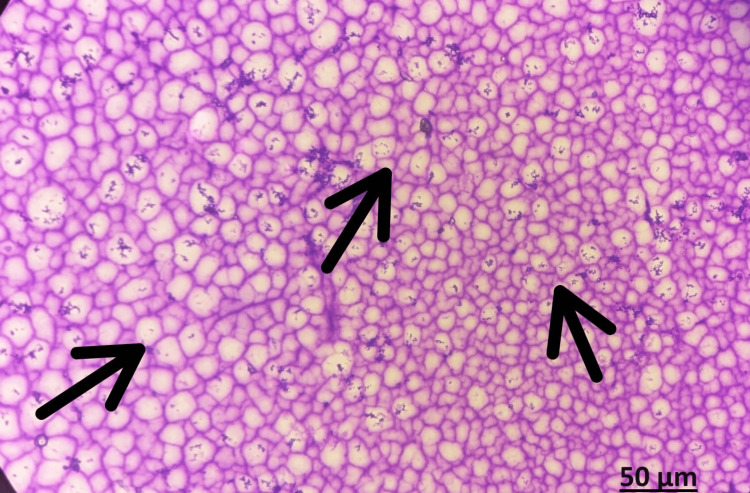
FNAC Showing cytomorphology suggestive of simple cystic lesion of Intraosseous ganglion cyst. Papanicolaou stains (PAP), Objective magnification: 40 X FNAC: fine needle aspiration cytology

The lesion's fine needle aspiration cytology (FNAC) reported cytomorphology that was indicative of an intraosseous ganglion cyst with a simple cystic lesion (Figure 3).

**Figure 4:**
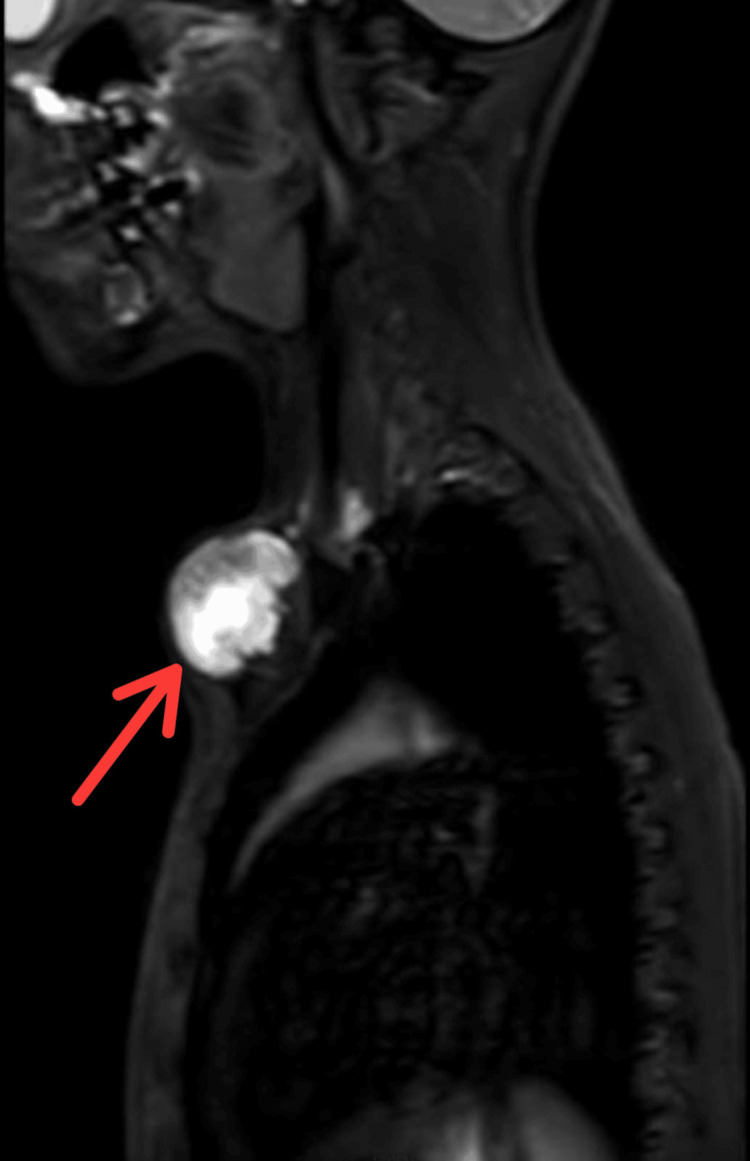
MRI sagittal view showing the extent of the lesion

**Figure 5:**
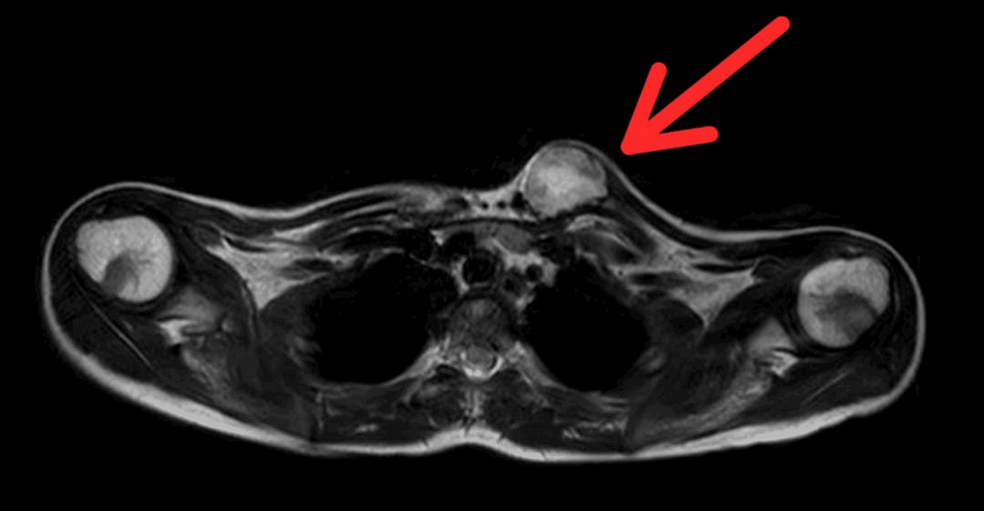
MRI showing the extent of the lesion

MRI revealed a well-defined expansile altered signal intensity mass lesion with a central necrotic area in the medial end of the left clavicle which caused cortical thinning and reported differentials as mostly neoplastic aetiology like sarcoma and less likely infective aetiology (Figure 4, 5).

**Figure 6:**
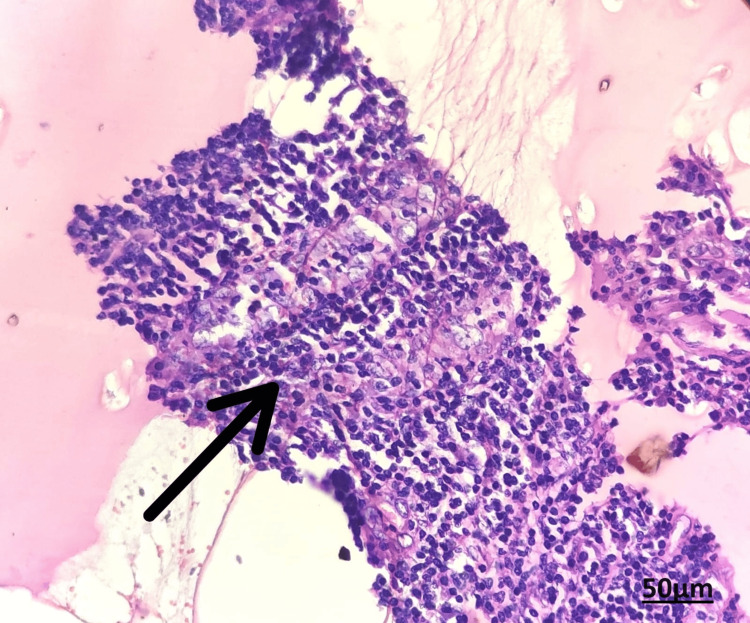
Section showing abundance of hyaline cartilage matrix, tumor cells are placed in sheets, nuclei is small and round, condensed chromatin Hematoxylin and eosin stain, Objective magnification: 40 X

**Figure 7:**
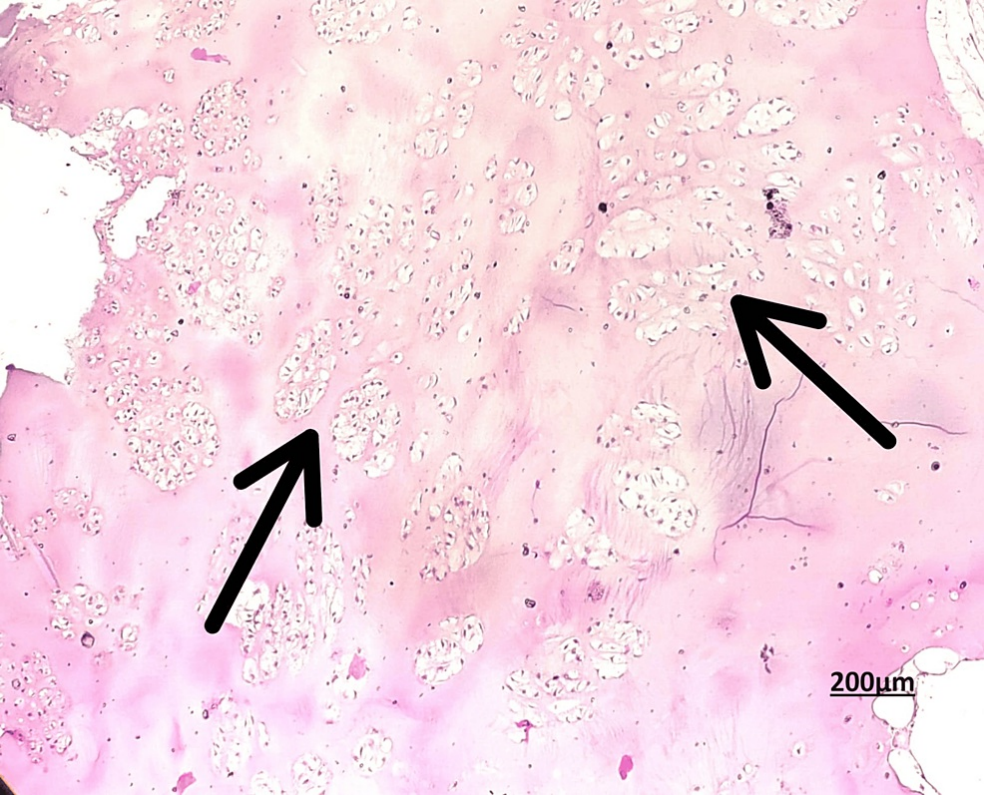
Section showing low N:C ratio and abundant eosinophilic cytoplasm Hematoxylin and Eosin stain, Objective magnification: 10X

Despite the aggressiveness implied by the radiological findings, the pathologist identified an intraosseous ganglion cyst. We proceeded to curettage the lesion and cauterise the cavity because the biopsy report of the sample did not show signs of chondrosarcoma. The excised tissues were sent for histopathology and the report came as enchondroma with no evidence of malignancy (Figures 6, 7).

**Figure 8:**
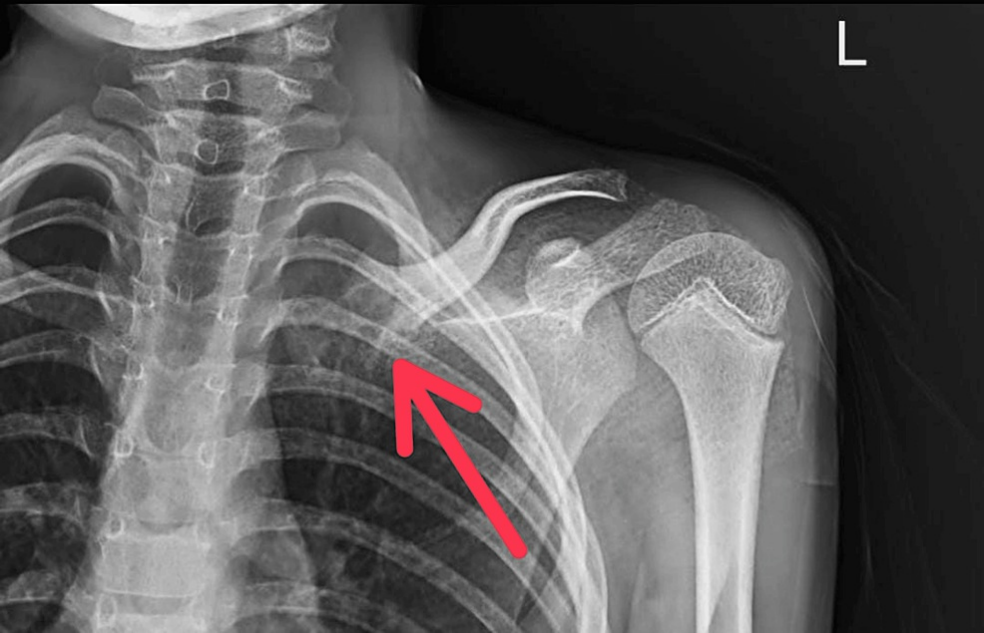
Post-operative X-ray after curettage of the lesion

Post-operative X-ray was found to be satisfactory (Figure 8).

After completing shoulder range of motion exercises as tolerated, the patient attained a complete range of motion six weeks later. Following that, the patient did not exhibit any symptoms and there were no signs or symptoms of distant metastasis or recurrence.

## Discussion

We reported the appearance of an uncommon enchondroma in a rare instance of a young child at the medial end of the clavicle, a site not usually associated with such events in long bones. The patient recovered completely with only curettage, even though the tumour suddenly grew larger.

Owing to their intramedullary origin, enchondromas are usually solitary; nevertheless, Ollier's illness or Maffucci's syndrome may be linked to multiple enchondromatosis [7]. They make up roughly 12-24% of benign bone tumours and 3-10% of all bone tumours [8]. Enchondromas are less common than osteochondromas in terms of frequency. The majority of patients are in the age group of 30-35 years, and gender does not show any particular susceptibility. They typically appear in the second decade of life. However, our patient did not fall into this vulnerable age bracket. Although certain features on radiographs can help diagnose enchondroma, there are other disorders that have characteristics that are similar to enchondroma. Thus, distinguishing enchondromas from chondrosarcoma, bone infarcts, bone islands, eosinophilic granuloma, fibrous dysplasia, Garre's sclerosing osteomyelitis, and isolated bone cysts is essential [9-11].

## Conclusions

Enchondromas usually occur in the diaphysis of long bones, but they can also occur in the metaphysis. Although these tumours are usually benign, when they exhibit aggressive traits in unusual sites, they might pose a diagnostic problem, leading surgeons to explore alternative options. The lesion may still be benign despite an abrupt rise in size and indications of intramedullary enlargement and cortical thinning. An intralesional curettage is frequently a successful treatment for such unusual enchondromatous lesions.
